# Innovative Biochemometric Approach to the Metabolite and Biological Profiling of the Balkan Thistle (*Cirsium appendiculatum* Griseb.), Asteraceae

**DOI:** 10.3390/plants10102046

**Published:** 2021-09-28

**Authors:** Dimitrina Zheleva-Dimitrova, Gokhan Zengin, Gunes Ak, Kouadio Ibrahime Sinan, Mohamad Fawzi Mahomoodally, Reneta Gevrenova, Vessela Balabanova, Alexandra Stefanova, Paraskev Nedialkov, Yulian Voynikov

**Affiliations:** 1 Department of Pharmacognosy, Faculty of Pharmacy, Medical University—Sofia, 2 Dunav Str., 1000 Sofia, Bulgaria; rgevrenova@pharmfac.mu-sofia.bg (R.G.); vbalabanova@pharmfac.mu-sofia.bg (V.B.); al_stefanova@abv.bg (A.S.); pnedialkov@pharmfac.mu-sofia.bg (P.N.); 2 Biochemistry and Physiology Research Laboratory, Department of Biology, Science Faculty, Selcuk University, Konya 42130, Turkey; gokhanzengin@selcuk.edu.tr (G.Z.); akguneselcuk@gmail.com (G.A.); sinankouadio@gmail.com (K.I.S.); 3 Department of Health Sciences, Faculty of Medicine and Health Sciences, University of Mauritius, Réduit 80837, Mauritius; f.mahomoodally@uom.ac.mu; 4 Department of Chemistry, Faculty of Pharmacy, Medical University—Sofia, Bulgaria 2 Dunav Str., 1000 Sofia, Bulgaria; voynikov_y@pharmfac.mu-sofia.bg

**Keywords:** *Cirsium appendiculatum*, UHPLC–HRMS, biochemometric, antioxidants, enzyme inhibitory activity, partial least-square discriminant analysis

## Abstract

The widespread genus *Cirsium* Mill. (Asteraceae) is renowned in traditional medicine. In the present study, an innovative biochemometric-assisted metabolite profiling of the flower heads, aerial parts and roots of *Cirsium appendiculatum* Griseb. (Balkan thistle) in relation to their antioxidant and enzyme inhibitory potential was developed. The workflow combines ultra-high-performance liquid chromatography–high-resolution mass spectrometry (UHPLC–HRMS) with partial least-square analysis to discriminate the herbal extracts and identify the most prominent biological activities. The annotation and dereplication of 61 secondary metabolites were evidenced, including 15 carboxylic (including hydroxybenzoic and hydroxycinnamic) acids and their glycosides, 11 acylquinic acids, 26 flavonoids and 9 fatty acids. All compounds were reported for the first time in the studied species. The root extract revealed the highest cupric and ferric reducing power (618.36 ± 5.17 mg TE/g and 269.89 ± 8.50 mg TE/g, respectively) and antioxidant potential in phosphomolybdenum (3.36 ± 0.15 mmol TE/g) as well as the most prominent enzyme inhibitory potential on *α*-glucosidase (0.72 ± 0.07 mmol ACAE/g), acetylcholinesterase (4.93 ± 0.25 mg GALAE/g) and butyrylcholinesterase (3.80 ± 0.26 mg GALAE/g). Nevertheless, the flower heads were differentiated by their higher metal chelating activity (32.53 ± 3.51 mg EDTAE/g) and total flavonoid content (46.59 ± 0.89 mgRE/g). The partial least-square discriminant and heat-map analysis highlighted the root extract as the most active and a promising source of bioactive compounds for the therapeutic industry.

## 1. Introduction

Profiling methods for the analysis of crude plant extracts have evolved into powerful tools for dereplication, quality assessment and metabolomics. This procedure enables recognition of known metabolites at the earliest stage of separation, avoiding the time-consuming and expensive isolation of common constituents. The most current metabolite profiling studies are performed with state-of-the-art high-resolution LC–MS tools that apply the high resolution of ultra-high-performance liquid chromatography (UHPLC) for the chromatographic resolution of isomers, and high-resolution MS methods for molecular formula assignment [1]. In particular, the hybrid quadrupole-orbitrap has high mass resolution and accuracy in MS non-targeted profilings of specialized (secondary) natural products in crude extracts [2]. In this context, biochemometrics approaches, which rely on the use of statistical modelling tools to correlate metabolite profiles with biological datasets, are very useful for assigning biological activity to a particular compound detected from complex mixtures.

The widespread genus *Cirsium* Mill. (thistle) is one of the biggest genera in Asteraceae family (subfamily: Carduoideae Cass. Ex Sweet, tribe: Cardueae Cass., subtribe: Carduinae (Cass.) Dumort, sect. *Cirsium*). It includes about 250 species spread throughout Europe, North Africa, East Asia, Central Asia, SW Asia and North and Central America [3,4]. Its species have been used for many years as a traditional herbal medicine. As the origin of the name suggests (“khirsos” in Greek means “swollen veins”), the genus *Cirsium* has been known for centuries for its usage against varicose diseases, to relieve pain [4]. According to the ethnopharmacological relevance, the species is also valued for the treatment of numerous ailments due to its diuretic, astringent, anti-inflammatory, anti-melanogenesis, anti-tumor and anxiolitic activities as well as its activity against nonalcoholic fatty liver disease [5,6]. Additionally, some *Cirsium* species are used as a food source. Receptacles of *C. spinosissimum* have been traditionally eaten similarly to artichoke leaves by alpine populations [7]. Moreover, *Cirsium* species are valuable to the honey industry as they produce a good supply of nectar and pollen. In the past decade, invasive exotic species, such as Eurasian thistles, present a major threat to sustained productivity and biodiversity in the United States, and different *Cirsium* species have been assessed for biological control as weeds [8].

The taxon is characterized by the presence of a large number of secondary metabolites such as phenolic acids, flavonoids, sterols, triterpenes, alkaloids and lignans [8,9]. The existence of flavones, flavonols and flavonones, free aglycones, their derivatives and glycosides has been proven [8,10,11]. The best-known and researched compounds in *Cirsium* are the flavonoids, which are found in all plant organs. Acacetin, apigenin, cirsimaritin, luteolin, quercetin, pectolinarigenin and their glycosides are among the most common flavonoids [4,6]. 

Given the notable amount of data on the traditional medicinal usage and therapeutic properties attributed to the *Cirsium* species, it is necessary to scientifically bioprospect poorly studied species belonging to this genus, such as *Cirsium appendiculatum* Griseb. (Balkan thistle) against significant human diseases such as diabetes, Alzheimer’s disease, atherosclerosis, etc. *C. appendiculatum* is an herbaceous perennial plant, up to 180 cm height, flowering from June to September. It is a Balkan endemic plant occurring in Turkey, Greece, Albania, North Macedonia, Serbia and Montenegro [12]. The species is distributed in the alpine zone at altitudes between 1000–2500 m asl and occurs in a wide range of open habitats such as meadows, forests and rivers [12]. 

An investigation of its health-promoting effects and applications in a variety of nutraceutical, pharmaceutical, medicinal and cosmetic areas represents interest, and the data will complete knowledge of the genus. Accordingly, a wide range of biological activities such as antioxidant activity and inhibitory effects against different enzyme classes were investigated.

In line with the new paradigm in pharmacognosy to obtain massive metabolite profiling of natural extracts for a rational prioritization of bioactive natural products [13], the present study was designed to investigate, for the first time, the phytochemical profile and biological activity of *C. appendiculatum* extracts. An innovative/efficient workflow based on the association of both UHPLC–HRMS and biochemometrics, using a combination of multiple statistical models (partial least-square discriminant and heat-map analyses) to target bioactive compounds from extracts was developed. 

## 2. Results and Discussion

**Figure 1:**
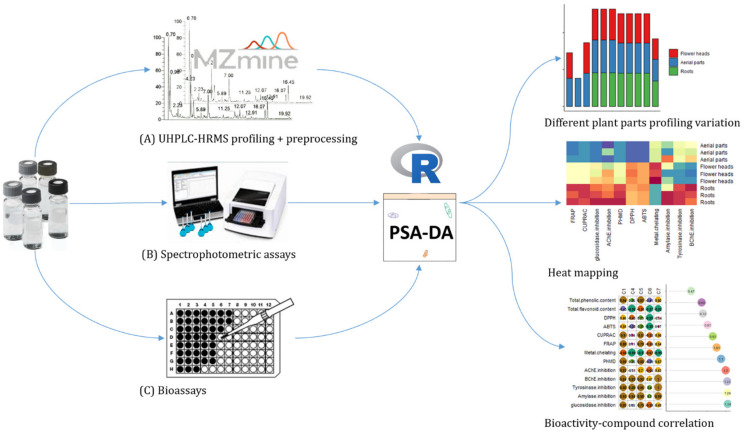
The complete workflow for the biochemometric approach. Samples (flower heads, aerial parts and root extracts) prepared at the same concentration are first injected into UHPLC–HRMS (**A**). Data are acquired using the data-dependent acquisition mode, then converted through MZmine 2 software processing. In parallel, spectrophotometric assays (**B**) and bioassays (**C**) are conducted to determine total phenolic and flavonoid contents and activity and information are tabulated. The final .csv files are then used for the generation of the biochemometic data by partial least-square discriminant analysis (PLS-DA) with R software. Finally, bioactivity mapping was performed.

The complete workflow combining UHPLC–HRMS with discriminant analysis of the chromatographic data and the biological potential is presented on Figure 1. 

### 2.1. UHPLC–HRMS Profiling of Specialized Natural Products in Cirsium appendiculatum Extracts

**Figure 2:**
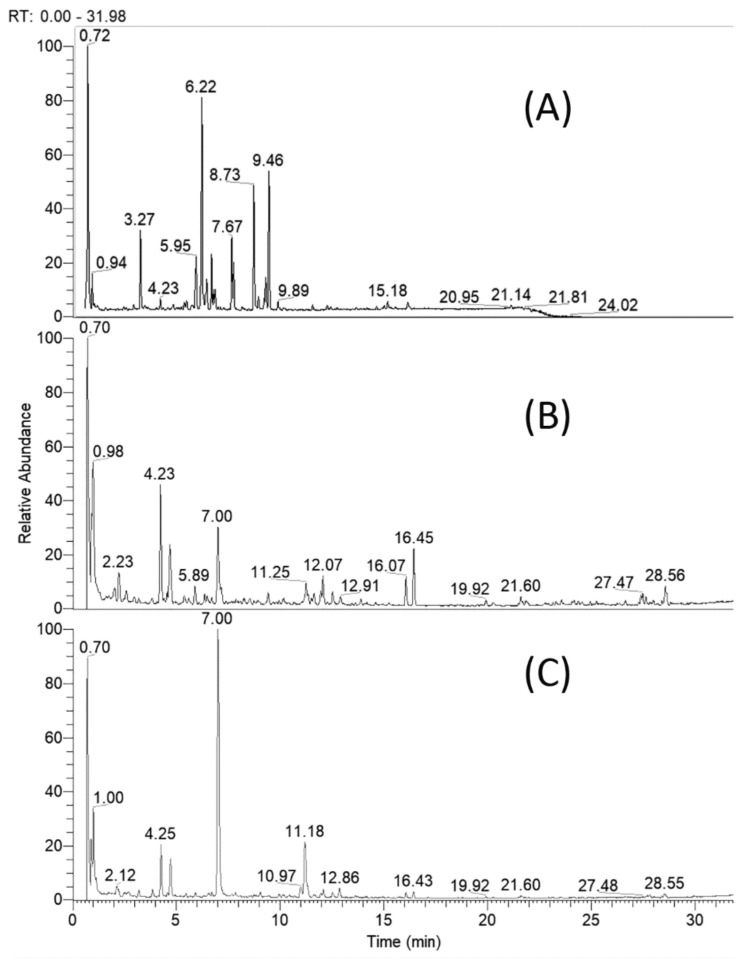
Total ion chromatogram of *Cirsium appendiculatum* extracts; (**A**) flower heads, (**B**) aerial parts, (**C**) roots.

Based on retention times, MS and MS/MS accurate masses and relative ion abundance, elemental composition, fragmentation patterns in MS/MS spectra, conformity to the simulated monoisotopic profiles and comparison with reference standards and literature data, a total of 61 specialized natural products were identified or tentatively annotated in *C. appendiculatum* extracts (Table 1). The total ion chromatograms (TIC) of the studied extracts are depicted in Figure 2.

In the biochemometric approach, peak areas (log (peak area)) for a data quantitative analysis were used. A designed graph clearly shows the differences of the phytochemical components’ distribution in the studied herbal extracts (Figure S1). Thus, flower heads contained more flavonoids compared to aerial parts and roots. Subsequently, a qualitative analysis was carried out. 

#### 2.1.1. Carboxylic (Including Hydroxybenzoic, Hydroxycinnamic and Acylquinic) Acids and Their Glycosides

Hydroxybenzoic (1–4) and hydroxycinnamic acids (5 and 8), their glycosides (9–15) and quinic acid (6) were identified based on a comparison with reference standards and literature data (Table 1 and Table S1). The tentative structure of compound 8 was deduced from fragment observation at *m/z* 197.045 [syringic acid-H]^−^ and 123.007 [syringic acid-H-CO_2_-2CH_3_]^−^, referring to the elimination of caffeoyl moiety (−162.05 Da) and eased its preliminary assignment as caffeoyl-syringic acid [2]. The MS/MS spectrum of 10 revealed a neutral loss of deoxyhexose (−146.09 Da), and diagnostic fragment ions for vanillic acid at m/z 167.034 [vanillic acid-H]^−^, 152.010 [vanillic acid-H-CH_3_]^−^ and 123.043 [vanillic acid-H-CO_2_]^−^, and were tentatively ascribed to vanillic acid *O*-deoxyhexoside (Table 1 and Table S1) [14]. In the same manner compounds 7 [15], 8 [16], 11 [17], 12 [18], 14 [19] and 15 [20] were tentatively annotated (Table 1 and Table S1). The isolation of 8 was not reported in the literature and therefore it could be referred to as “unknown”.

The acylquinic acid dereplication was based on conformity with the structure-diagnostic hierarchical keys for chlorogenic acid identification proposed by Clifford et al. [21] and later developed by Jaiswal et al. [22], as well as literature data acquired by hybrid Q-Orbitrap mass spectrometry [2]. Thus, six mono-acylquinic (16–21), four di-acylquinic (22–25) and one tri-acylquinic (26) acids was annotated in the studied extracts.

#### 2.1.2. Flavonoids, Flavones and Flavonols

The aglycones apigenin (27), luteolin (30) and quercetin (33) were deduced from the Retro-Diels-Alder (RDA) cleavages ^1,3^A^−^ at *m/z* 151.002, ^1,3^B^−^ at *m/z* 117.033 (27) and 133.028 (30), ^0,4^A^−^ at *m/z* 107.012, ^1,2^A^−^ at *m/z* 178.998 (33) and ^1,2^B^−^ at *m/z* 121.028 (33) (Table 1 and Table S1) [2]. The fragmentation pathways of 38, 40 and 50 showed neutral mass losses of deoxyhexose (146.059 Da), hexose (162.053 Da) and rutinose (308.112 Da). Consequently, the aglycone was registered at *m/z* 285.041 together with the radical aglycone at *m/z* 284.033 [Y-H-]^−•^, as usually seen in flavonoids substituted at the 3-position, i.e., flavonol 3-*O*-glycosides. Fragment ions at *m/z* 255.030 and 227.035 resulting from neutral losses of CH_2_O and CO, respectively, being present in high abundance corroborated kaempferol [2]. MS/MS spectra of 39 and 42 demonstrated neutral mass losses of hexuronic acid (176.033 Da) together with base peaks at *m/z* 269.045 and 285.0403. Accordingly, 39 and 42 could be associated with apigenin *O*-hexuronide and luteolin *O*-hexuronide. Compounds 27, 30, 37, 40 and 41 were unambiguously identified by a comparison with retention times in LC-MS and fragmentation fingerprints of the reference standards.

#### 2.1.3. Methoxylated Flavonoids

**Figure 3:**
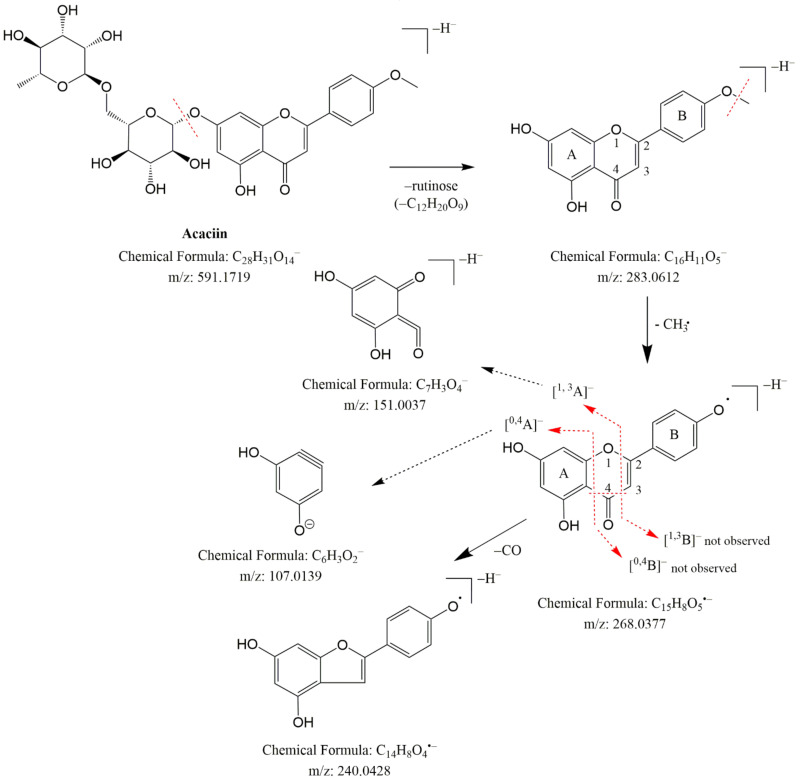
Fragmentation pathway of acaciin (49).

MS/MS spectrum of 34 revealed fragment ions at *m/z* 298.048 [M-H-•CH_3_]^−^ and 283.025 [M-H-2•CH_3_]^−^, indicating a subsequent loss of two methyl radicals. A series of fragment ions resulting from neutral losses were registered at *m/z* 255.029 [M-H-2•CH_3_-CO]^−^, 227.034 [M-H-2•CH_3_-2CO]^−^, 211.039 [M-H-2•CH_3_-CO-CO_2_]^−^ and 183.044 [M-H-2•CH_3_-2CO-CO_2_]^−^ (Table 1 and Table S1). A methoxy group at C-6 (ring A) and another one in ring B were deduced from the lack of the initial RDA ions ^1,3^A^−^ (181.014) and ^1,3^B^−^ (147.045). In addition, the neutral losses of 16 Da (CH_4_), 14 Da (CH_2_) and 28 Da (CO) afford fragment ions in the low mass range at *m/z* 136.988 (^1,3^A^−^-CH_4_-CO) and *m/z* 117.028 (^1,3^B^−^-CH_2_) (Table 1 and Table S1, Figure 3).

These data corresponded to the Justesen key for methoxylated flavonoid dereplication [23] and the detailed analysis of the fragmentation pathway of methoxylated flavonoids done by Ren et al. [24]. Thus, 34 was identified as pectolinarigenin (Table 1). By analogy with 34, 35 gave fragment ions at *m/z* 136.986 (^1,3^A^−^-CH_2_-H_2_O-CO) and ^1,3^B^−^ at *m/z* 133.028, indicating a methoxyl group in the A ring. Compound 35 was ascribed to nepetin, previously isolated from *Cirsium* species [8]. The isobaric pair 28/29 afforded [M-H]^−^ at *m/z* 283.061 and fragment ions at *m/z* 268.037 [M-H-•CH_3_], 240.043 [M-H-•CH_3_-CO]^−^and 239.034 [M-H-CO_2_]^−^, indicating methoxylated flavonoids. A free hydroxyl group in the B ring of 28 was deduced from the RDA fragment ion at *m/z* 117.033 (^1,3^B^−^), while a diagnostic ion at *m/z* 165.349 (^1,3^A^−^), corresponding to the methoxyl group, was deduced in the A ring. These data are consisted with Justesen [23] and 28/29 were ascribed to genkwanin (4′,5-dihydroxy-7-methoxyflavon) and acacetin (5,7-dihydroxy-4′ methoxyflavon), respectively (Table 1 and Table S1).

Two peaks (31 and 32) produced the same [M-H]^−^ at *m/z* 299.056 and a fragment ion at *m/z* 284.032 [M-H-•CH_3_]^−^ (Table 1 and Table S1). Compound 32 gave diagnostic ions at *m/z* 256.035 [M-H-•CH_3_-CO]^−^, 151.002 (^1,3^A^−^) and 107.012 (^0,4^A^−^), indicating that the methoxy group is situated in the B ring. Compound 31 yielded a relevant fragment ion at *m/z* 136.988 (^1,3^A^−^-CH_4_-CO), corresponding to a methoxyl group at C-6 in the A-ring. Thus, 31 and 32 could be related to hispidulin and diosmetin, respectively (Table 1 and Table S1) [23]. In the same manner, 36 afforded diagnostic ions at *m/z* 314.043 [M-H-•CH_3_]^−^, 299.019 [M-H-2•CH_3_]^−^, 271.025 [M-H-2•CH_3_-CO]^−^ and 227.035 [M-H-2•CH_3_-CO-CO_2_]^−^ together with RDA fragments at *m/z* 161.023 [^1,3^A^−^-CH_4_-H_2_O]^−^ and 151.002 [^1,3^A^−^-CH_4_-CO]. Accordingly, 36 could be associated with cirsiliol, previously determined in *Cirsium* species [8].

By analogy to flavones and flavonols, the glycosides of methoxylated flavonoids 43–49 and 51–52 were ascribed. MS/MS spectra of 49 and 52 revealed base peaks corresponding to the simultaneous loss of hexose and deoxyhexose. The aglycone of 49 showed a fragmentation pathway similar to acacetin (29) [23], while 52 corresponded to pectolinarigenin (34). Thus, 49 and 52 were identified as rutinosides acaciin and pectolinarin, respectively. The identification of 31, 49 and 52 was confirmed by comparison with reference standards (Table 1 and Table S1).

#### 2.1.4. Free Fatty Acids

In (−) ESI-MS/MS of 53 (C_9_H_16_O_4_), [M-H]^−^ at *m/z* 187.096, the subsequent and concomitant losses of H_2_O (−18) and CO_2_ (−44) yielded several characteristic ions at *m/z* 166.990 [M-H-H_2_O]-, *m*/*z* 143.106 [M-H-CO_2_]^−^ and 97.064 [M-H-2CO_2_]^−^, and a base peak at *m/z* 125.095 ([M-H-CO_2_-H_2_O]^−^. Based on comparison with the literature data, 53 could be related to the saturated dicarboxylic acid-nonanedioic acid (azelaic acid) [25] (Table 1 and Table S1). Compound 55 demonstrated a similar fragmentation pathway. However, 55 gave fragment ions at *m/z* 143.070 [C_7_H_11_O_3_]^−^, 113.095 [C_7_H_1__3_O]^−^ and 59.012 [C_2_H_3_O_2_]^−^, corresponding to a presence of the hydroxyl group at C-3. Thus, 55 was ascribed to 3-hydroxylazelaic acid (Table 1 and Table S1). Compound 54 differs from 55 by one CH_2_ group and was tentatively identified as 3-hydroxyoctandioic acid (3-hydroxysuberic acid) (Table 1 and Table S1). Based on a comparison between metabolites’ AUC, compound 54 was found to be the major compound in *C. appendiculatum* roots.

In the (−) ESI-MS/MS spectrum of 56 (C_12_H_20_O_4_), a base peak at *m/z* 183.138 [M-H-CO_2_]^−^ and a fragment ion at *m/z* 165.127 (C_11_H_17_O) indicated the presence of double bond at C-2. Thus, the compound was identified as dodec-2-endioic acid (traumatic acid). Similarly, 57–61 were identified as polyunsatured fatty acids [26] (Table 1 and Table S1).

### 2.2. Total Content of Phenolics and Flavonoids

Polyphenols and their biological properties are one of the most attractive topics in the natural sciences. Nowadays, humanity needs to substitute synthetic compounds with natural ones. This means safe and alternative raw materials need to be found [27]. In this sense, total phenolic and flavonoid content is considered a first insight in evaluating plant extracts. Thus, the total amount of these biocompounds in tested extracts were determined by using spectrophotometric methods (Table 2). Root extract was found to have the highest content of phenolics (143.62 mgGAE/g), followed by flower heads and aerial parts (71.75 ± 1.47 mgGAE/g and 26.02 ± 1.49 mgGAE/g, respectively).

Regarding total flavonoids, the values are in the following order: flower heads (46.59 mg RE/g) > roots (3.99 mg RE/g) > aerial parts (2.64 mg RE/g). Hence, flavonoids represent about 50% of the total phenolic components in flower head extract. According to a literature survey, different levels of total bioactive compounds in *Cirsium* species were observed [28,29]. These differences could be linked to the habitat of the studied plant, climate conditions or extraction procedures/solvents. However, in past years, the utilization of spectrophotometric methods for total content of bioactive compounds has led to some concerns, and these methods are not used by most scientists anymore [30]. Thus, plant matrices are very complexed and phenolics as well as other components such as peptides could be reacting with Folin’s reagent. Finally, the exact quantity of bioactive constituents has to be confirmed by chromatographic techniques such as LC–MS/MS, NMR and Q–TOF-MS analysis.

### 2.3. Antioxidant Properties

In the present study, *C. appendiculatum* extracts were tested for antioxidant potential (Table 2). DPPH^•^ and ABTS^•+^ were used to evaluate radical scavenging ability. The root (97.95 mg TE/g for DPPH^•^ and 224.59 mg TE/g for ABTS^•+^) and flower head extracts (101.79 mg TE/g for DPPH^•^ and 224.57 mg TE/g for ABTS^•+^) displayed the strongest abilities. The aerial parts extract had the lowest capacity in both scavenging assays. The reduction abilities of the studied herbal extracts were evaluated using the CUPRAC and FRAP methods, and they are closely linked to the electrohern-donating potential of the extracts. The most prominent reduction ability was observed in the roots, followed by the flower heads and aerial parts. In terms of reduction of Mo (VI) in the phosphomolybdenum (PHMD) assay, the extracts can be ranked as follow: roots > flower heads > aerial parts. In general, the antioxidant data showed the same trends in total phenolic levels. This fact was supported by several authors who reported a strong correlation between total phenolics and radical scavenging and reducing abilities [31]. However, the metal chelating method based on the binding of transition metals by phytochemicals did not correlate with the other antioxidant methods. Regarding the metal chelating assay, the best ability was registered in the flower head extract (32.53 mg EDTAE/g), while the root sample was not active. 

### 2.4. Enzyme Inhibitory Effects

Nowadays people are battling noncommunicable illnesses like diabetes mellitus, obesity and Alzheimer’s. In particular, changes in lifestyle and dietary preferences increase the risk of these diseases. In the course of scientific study, some enzymes can be valuable tools against these health problems [32]. This approach is known as the enzyme inhibitory theory, in which some enzymes play a role in the pathologies of these diseases. For example, amylase and glucosidase are the main targets for controlling blood sugar levels in diabetes patients [33]. In addition, lipase is the main target for controlling obesity. In the present study, the inhibitory effects of different enzyme classes were investigated. Acetylcholinesterase (AChE) and butyrylcholinesterase (BChE) belong to the same structural class of proteins, the esterase/lipase family, amylase and glucosidase are hydrolases, while tyrosinase is an oxidoreductase enzyme. Given this information, several compounds have been chemically produced as inhibitors. Although they are accepted as effective agents in the control of global health problems, concerns have been raised regarding some disturbances to wellbeing [34]. In this regard, plants are considered to be the most important and richest natural source of enzyme inhibitors such as alkaloids, phenolic acids and terpenoids. Recent studies have shown that some plants and their constituents showed promising inhibitory effects on key enzymes that have been linked to significant health problems [35]. Hence, the enzyme inhibitory properties of *C. appendiculatum* extracts were examined (Table 3). In both AChE and BChE inhibition assays, the root extract exhibited the highest inhibitory values (4.93 mg GALAE/g and 3.80 mg GALAE/g, respectively). The lowest abilities were recorded for aerial parts and flower head samples. Regarding tyrosinase inhibition ability, all extracts showed inhibitory effects, and the values ranged from 97.78 to 127.99 mg KAE/g in the following order: flower heads < aerial parts < roots. All the tested herbal extracts had similar amylase inhibition capacity (*p* > 0.05), while the strongest glucosidase ability was observed in root extract (0.72 ± 0.07 mmolACAE/g). In addition, the aerial parts sample was not active in glucosidase.

The data could be related to the different chemical components which were identified in the tested extracts (Table 1). For example, flavonoids and acylquinic acids dominate in the chemical profiles. Several flavonoids, including quercetin, luteolin and apigenin have been described as significant enzyme inhibitors [35]. In addition, chlorogenic acid and its derivatives are known to be important neuroprotectors and antidiabetic agents. Some data on the enzyme inhibitory properties of *Cirsium* species have been found [29]. Thus, the obtained data in the current study can be a valuable contribution to the development of new active substances agents against Alzheimer’s disease, and diabetes and its complications. The methoxylated flavone derivatives pectolinarin and its aglycon pectolinarigenin are important for the pharmacological activity of the genus [8]. The aforementioned are responsible for the control of diabetes and other metabolic disorders, and are prominently represented in the chemical composition of *C.* *japonicum* [36]. Pectolinarin and its aglycone individually significantly reduce glucose levels, but the strongest antidiabetic effect is achieved when they are combined [36].

### 2.5. Supervised Multivariate Analysis

**Figure 4:**
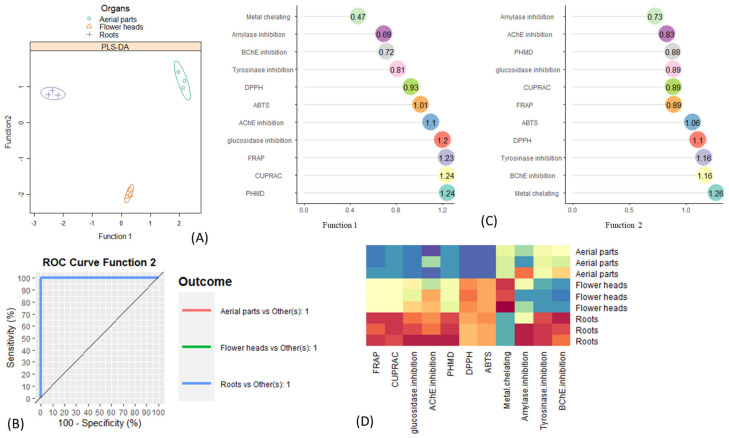
Partial least-square discriminant analysis graphical outputs on the biological activities (with three replications). (**A**) The sample plots. (**B**) Roc curve and AUC averaged using one-vs-all comparisons. (**C**) The most discriminant biological activities identified though VIP score calculation. (**D**) Heatmap displaying variation of biological activities between the three organs. Red color = high activity. Blue color = low activity.

Taking into account the variation in quality and quantity of chemical compounds within the different parts of plants, we postulated that there would be variation in certain biological activities between the three studied parts of *C. appendiculatum*. In order to verify this hypothesis, we performed a supervised multivariate analysis. Partial least-square discriminant analysis (PLS-DA) is a commonly used technique for achieving classification models for sample discrimination and for identifying and excluding the less discriminant variables to include only the variables of interest. PLS-DA is particularly suitable for dealing with a much greater number of variables than observations and with multicollineality between those variables. Hence, this statistical approach was employed in an attempt to compare the different parts studied by considering all evaluated biological activities together. By referring to the results available in Figure 4A, a clear segregation of the three parts was observed, with excellent model robustness. Indeed, the sensitivity and specificity of the model when using the first two function were 100%, which proved the accuracy of the PLS-DA model based on the biological activities for discriminating the three parts (Figure 4B). Afterwards, recourse to the variable selection method VIP (variable importance in projection) helped to identify the most discriminant biological activities responsible for the observed segregation (Figure 4C). As part of the current study, biological activities having a VIP score above 1.2 were considered the most significant in parts separation. In this sense, the four biological activities (PHMD, CUPRAC, FRAP and glucosidase inhibition) having a VIP score above 1.2 on function 1 of the PLS-DA allowed a separation of the roots from the flower heads and aerial parts. On the other hand, only metal chelating (with VIP = 1.26 on function 2 of PLS-DA) was the top biological activity which allowed a differentiation of the flower heads from both the roots and the aerial parts. In viewing the heat map, we noticed that among the studied herbal extracts, overall, the roots displayed the most potent biological activity and thus could be a promising source of biocompounds for the therapeutic industry (Figure 4D). The variability of the biological activities between the samples was corroborated by previous studies reporting that the biological activities of the given species may fluctuate between the organs due to the heterogeneous distribution of bioactive compounds between the organs [37]. For example, several investigations reported that condensed tannins exist widely in stems, while flavonoids are typically accumulated in flower tissues. Moreover, according to Trabelsi et al. [38], the heterogeneous distribution of bioactive compounds between the organs may be linked to the physiological roles of these different organs. That the highest number of biological activities occurred in the roots was not surprising given the abundance of total phenolic content compared with the flower heads and aerial parts. According to Fernandez et al. [39], the roots’ bioactive compounds may be produced in response to soil-borne pathogens, i.e., insects and microbes. Additionally, the production of these compounds may be also due to indirect defense mechanisms against the root feeders of the other plants in the same environment.

### 2.6. Relationship between Chemical Compounds and Biological Activities

**Figure 5:**
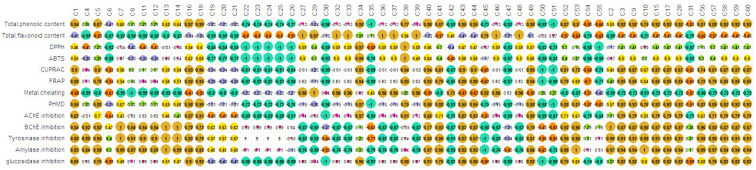
Correlation between evaluated biological activities and identified natural products. Brown color: *r* = [0.71; 1]; yellow color: *r* = [0.42; 0.70]; green color: *r* = [0.16; 0.41]; pink color: *r* = [−0.14; 0.15]; purple color: *r* = [−0.43; −0.15]; orange color: *r* = [−0.71; −0.44]; dark green color: *r* = [−1; −0.72].

The contribution of the identified compounds to biological activities was assessed through Pearson’s correlation. The results, depicted in Figure 5, showed several significant positive correlations between multiple biocompounds and various biological activities (*r* > 0.7). This could mean that different biocompounds were involved in the same biological activity as well as that a biocompound was involved in different biological activities. Thus, regarding the antioxidant assays, a synergistic or additive interaction is expected likely to occur between various compounds such as caffeic acid, protocatechuic acid, apigenin and quercetin, which have been proved to have excellent antioxidant properties. The strong antioxidant and enzyme-inhibitory activity of the root extract could be due to the presence of azelaic acid (53) and 3-hydroxyazelaic acid (55). Azelaic acid exhibits antioxidant and anti-inflammatory activity and is characterized by the influence of a number of enzymes, including tyrosinase, a key enzyme in melanogenesis. Azelaic acid affects inflammation by inhibiting the formation of free radicals (produced by neutrophils) and reducing the effects of reactive oxygen species, as well as inhibiting the peroxidation of arachidonic acid [40]. Phenolic compounds, widespread in *Cirsium*, determine the antioxidant activity of its species. Studies have shown that compounds such as cirsimaritin, hispidulin and cirsimarin are of major importance for the inhibitory ability of *C. japonicum*. Significant radical scavenging activity was observed as well as a protective effect against the lipid peroxidation of cell membranes, comparable to the antioxidant activity of vitamin E [9].

Hydroxybenzoic acids and their derivatives 1, 3, 9 and 15 and acylquinic acids 16−18 together with flavonoids 40, 43, 44 and 49 could be considered the potential key constituents in the phenolic level. Flavon aglycones (27, 32, 36) and glycosides (39, 46, 49) together with quercetin (33) could be the most likely contributors to the flavonoid level. Herein, two flavonoids (34 and 38) were the most potent radical-scavenging flavonoids in DPPH^•^ and ABTS^•+^ assays. The results indicated that the aforementioned flavonoids along with 40, 43, 44 and 49 contributed in reducing the power and the effect of the flavonoids that appeared to be higher than those of acylquinic acids, represented mainly by 16 and 18. In contrast, a series of flavon-hexuronides, 39, 45 and 48 and hexosides 37 and 46 accompanied by the flavon aglycons 27, 29, 32 and 36 were considered to have potential key metal chelating activity. Previous investigations revealed stronger antioxidant activity of 3,5-dicaffeoylquinic acid (IC50 2.62 μg/mL for DPPH and 2.76 ± 0.65 mM TE/mg for FRRP) than chlorogenic acid and (IC50 7.24 μg/mL for DPPH^•^ and 2.21 ± 0.14 mM TE/mg for FRRP) [41]. In addition, DPPH^•^ radical scavenging of luteolin (IC_50_ of 53 µg/mL) was found to be similar to those of Trolox (36.9 µg/mL). Moreover, luteolin is several times stronger than apigenin (even 20 times at 2.5 µg/sample) [42]. Eucomic acid (7), one of the main components in the aerial parts and roots of *C. appendiculatum*, has a proven antioxidant effect.

As a phenolic compound, it is able to easily donate hydrogen atoms to free radicals, thus blocking the chain propagation step occurring in the oxidation process (H-atom transfer mechanism). On the other hand, 7 can give an electron to the free radical, turning it into a cation radical (a mechanism of free one-electron transfer). In addition, deprotonated carboxyl groups behave like electron-donor groups, thus contributing to H-atom transfer and radical scavenging activity by electron donation [43]. 

In the same line, several compounds likely synergistically bind to the studied enzymes, i.e., tyrosinase, amylase, glucosidase as well as cholinesterase. Concerning AChE inhibitory activity, the analysis indicated that two acylquinic acids (16 and 18), two flavonoids (34 and 38) and two hydroxybenzoic acid-pentosides (10 and 15) intensively acted on the enzyme. Phenolic acids 2, 4 and 5 and hydroxybenzoic acid-hexosides 11–13 together with pectolinarin (52) and fatty acids 53 and 56–60 can pronouncedly inhibit BChE. Previously, acaciin and acacetin 7-*O*-β-D-galactopyranoside were found as the compounds responsible for the AChE inhibition. The relationship between structure and activity has revealed that the presence of methoxy groups at C-4′ in the B ring and a sugar at O-7 in ring A appeared to be essential for the inhibition of AChE [44]. In our study, eucomic acid (7), leonuriside A (14) and rutonosides of hispidulin and pectolinarigenin (51, 52) caused the most potent inhibition of tyrosinase. Interestingly, the aforementioned compounds were found among the inhibitors of α-amylase. This is more plausible since Yu et al. [45] demonstrated that quercetin, ferulic acid and cinnamic acid synergistically inhibit tyrosinase. *p*-Coumaroyl- and caffeoylquinic acid (16 and 18) together with pectolinarigenin (34), kaempferol 3-*O*-glucoside (40) and acaciin (49) were involved in the inhibition of α-glucosidase. In addition, pectolinarigenin and pectolinarin possess anti-inflammatory activity and they may inhibit eicosanoid formation in inflammatory lesions [46]. Azelaic acid (53), together with other dicarboxylic acids (C9–C12), has been shown to inhibit the enzyme tyrosinase, thereby directly affecting melanin biosynthesis. The effect of azelaic acid on the progression of malignant melanomas has been proven. Studies on cell cultures of keratinocytes, melanocytes and melanoma cells have shown the action of this acid as an inhibitor of DNA synthesis and cell proliferation. Azelaic acid reversibly inhibits thioredoxin reductase, a membrane-bound enzyme that is responsible for reducing free radicals in the epidermis and regulating melanin biosynthesis [47].

Consequently, it can be deduced from the findings that all observed biological activities depend on the concentration, the structure and the interaction between different bioactive compounds.

## 3. Materials and Methods

### 3.1. Plant Material

The plant material (whole samples of *Cirsium appendiculatum* with roots, aerial parts and flower heads) were collected on Vitosha Mt., “Zlatni mostove” locality at 1404 m a.s.l. (42.41° N 23.23° E), during the full flowering stage in July 2018. The plant was identified by Reneta Gevrenova (Assoc. Prof. in the Department of Pharmacognosy, Faculty of Pharmacy, Medical University—Sofia) according to Stoyanov et al. [12]. A voucher specimen was deposited at the Herbarium Facultatis Pharmaceuticae Sophiensis, Medical University—Sofia, Bulgaria (Voucher specimen № 11 615). Then, seven plant samples were separate into roots, aerial parts (stems and leaves) and flower heads dried at room temperature to constant weight. The dried plant materials were powdered using a laboratory mill.

### 3.2. Sample Extraction 

Air-dried powdered roots, aerial parts (stems and leaves), and flower heads (15 g) were extracted with 80% MeOH (1:20 *w*/*v*) by sonication (80 kHz, ultra-sound bath Biobase UC-20C) for 15 min (×2) at room temperature. Then, the extracts were concentrated in vacuo and lyophilized (lyophilizer Biobase BK-FD10P) to yield crude extracts as follows: flower heads 3.54 g, aerial parts 2.82 g and roots 2.14 g. The lyophilized extracts (1 mg) were dissolved in 80 % methanol (10 mL). An aliquot (2 mL) of each extract solution was filtered through a 0.45 μm syringe filter disc (Polypure II, Alltech, Lokeren, Belgium) and subjected to UHPLC–HRMS analyses.3.3. Chemicals

Acetonitrile and formic acid for LC–MS, and HPLC grade methanol were purchased from Fisher Scientific (Hampton, NY, USA).

The authentic standards used for compound identification were obtained as follows: gentisic acid, vanillic acid, protocatechuic acid, quercetin, luteolin, apigenin, genkwanin, apigenin 7-*O*-glucoside, kaempferol 3-*O*-glucoside, luteolin 7-*O*-glucoside and kaempferol 3-*O*-rutinoside, from Extrasynthese (Genay, France); caffeic acid, neochlorogenic acid, 3,4-dicaffeoylquinic acid, 1,5-dicaffeoylquinic acid and hispidulin were supplied from Phytolab (Vestenbergsgreuth, Germany); chlorogenic acid acaciin and pectolinarin were purchased from Sigma-Aldrich (St. Louis, MO, USA).

### 3.3. UHPLC–HRMS

Separation was achieved on a reversed phase column Waters Cortecs C18 (2.7 µm, 2.1 mm × 100 mm), column maintained at 40 °C. The binary mobile phase consisted of A: 0.1% formic acid in water and B: 0.1% formic acid in acetonitrile. The run time was 33 min. The following gradient was used: the mobile phase was held at 5% B for 1 min, gradually turned to 30% B over 19 min, increased gradually to 50% B over 5min, increased gradually to 70% B over 5 min and finally increased gradually to 95% over 3 min. The system was then turned to the initial condition of 5% B and equilibrated over 4 min. The flow rate and the injection volume were set to 300 µL/min and 1 µL, respectively. The effluents were connected on-line with a Q Exactive Plus Orbitrap mass spectrometer, where the compounds were detected.

Mass analyses were carried out on a Q Exactive Plus mass spectrometer (Thermo Fisher Scientific, Inc., Waltham, MA, USA) equipped with a heated electrospray ionization (HESI-II) probe (Thermo Scientific). The tune parameters were as follows: spray voltage −2.5 kV; sheath gas flow rate 38 AU; auxiliary gas flow rate 12 AU; spare gas flow rate 0 AU; capillary temperature 320 °C; probe heater temperature 320 °C; and S-lens RF level 50. Acquisition was acquired at the full-scan MS and Data Dependent-MS^2^ modes. Full-scan spectra over the *m/z* range 100 to 1500 were acquired in negative ionization mode at a resolution of 70,000. Other instrument parameters for Full MS mode were set as follows: automatic gain control (AGC) target 3e6, maximum injection time (IT) 100ms, number of scan ranges 1. For the DD-MS^2^ mode, the instrument parameters were as follows: microscans 1, resolution 17,500, AGC target 1e5, maximum IT 50ms, MSX count 1, Top5, isolation window 2.0 *m/z*, stepped normalized collision energy (NCE) 10, 20, 60 eV. Data acquisition and processing were carried out with Xcalibur 4.0 software (Thermo Scientific, Inc.). 

### 3.4. Total Phenolics and Flavonoid Contents Determination

Total flavonoid and phenolic contents in the studied extracts were determined spectrophotometrically as described by Zengin and Aktumsek [48]. Standard compounds were used to express the obtained results (rutin (mg RE/g) for TFC and gallic acid (mg GAE/g) for TPC).

### 3.5. Biological Activities Determination 

The metal chelating, phosphomolybdenum method (PHMD), ferric reducing antioxidant power (FRAP), cupric reducing antioxidant capacity (CUPRAC), 2,2′-azino-bis (3-ethylbenzothiazoline-6-sulphonic acid) (ABTS^•+^) and 2,2-diphenyl-1-picrylhydrazyl (DPPH^•^) scavenging activities of the extracts were evaluated as described by Grochowski et al. [49]. Positive controls were ethylenediaminetetraacetic acid (EDTA) for metal chelating and trolox (TE) for the other methods. The possible enzymatic inhibitory activities of the extracts against acetylcholinesterase (AChE), butyrylcholinesterase (BChE) (by Ellman’s method), tyrosinase, α-amylase and α-glucosidase were assessed using bioassays [49].

### 3.6. Data Processing

Metabolite profiling using MZmine 2 software was applied to the UHPLC–HRMS raw files of the studied *C. appendiculatum* extracts.

#### 3.6.1. Univariate Analysis

All data were presented as mean ± SD, and the statistical analyses were performed by R software v. 3.6.1. The differences in the biological activities were assessed by using one-way ANOVA (*p* < 0.05).

#### 3.6.2. Supervised Multivariate Analysis

Taking into account all the evaluated biological activities together, PLS-DA was conducted to discriminate the plant organs. The area under the receiver operating characteristic curve (ROC AUC) was plotted to identify the optimal number of functions of a model, allowing for better discrimination between the observations as well as the goodness of the model. The VIP score was calculated to identify the most discriminant biological activities. 

#### 3.6.3. Pearson Correlation

A correlation map was created to display the relationships between the bioactive compounds and the observed biological activities.

## 4. Conclusions

In conclusion, an integrated approach combining the UHPLC–HRMS profiling of specialized natural compounds of the Balkan thistle (*Cirsium appendiculatum*) with discriminant analysis of biological activity was developed. An Orbitrap-based mass spectrometry strategy was used for the annotation and dereplication of 61 specialized natural products including carboxylic, hydroxybenzoic, hydroxycinnamic and acylquinic derivatives, methoxylated flavonoids and fatty acids; all compounds are reported for the first time in the species. The partial least-square discriminant and heat-map analysis allowed an overview of the specialized natural products, bringing insight into herbal extract-specific patterns. The proposed biochemometric approach allowed the determination of the contribution of the identified metabolites from the extracts on biological activities without the isolation of individual compounds and it could be useful in the phytopharmacological investigation of poorly studied plants. The obtained results highlighted the potential benefits of *C. appendiculatum* root extract for the antioxidant response and enzyme objectives that are associated with worldwide health problems.

## Figures and Tables

**Table 1:** Specialized natural products in *Cirsium appendiculatum* extracts.

№	Identified/Tentatively Annotated Compound	Molecular Formula	Exact Mass [M-H]^−^	t_R_ (Min)	Δ ppm	Distribution	Level of Identification (CAWG)
**Carboxylic (including hydroxybenzoic and hydroxycinnamic) acids**							
**1.**	protocatechuic acid ^a^	C_7_H_6_O_4_	153.0179	2.15	−7.986	1, 2	1
**2.**	dihydroxybenzoic acid	C_7_H_6_O_4_	153.0181	3.44	−8.182	2, 3	2
**3.**	gentisic acid ^a^	C_7_H_6_O_4_	153.0179	3.86	−9.685	2	1
**4.**	vanillic acid ^a^	C_8_H_8_O_4_	167.0338	4.77	−6.837	1, 2, 3	1
**5.**	caffeic acid ^a^	C_9_H_8_O_4_	179.0340	3.51	−5.317	1, 2, 3	1
**6.**	quinic acid	C_7_H_12_O_6_	191.0551	3.18	−5.032	1, 2, 3	2
**7.**	eucomic acid	C_11_H_12_O_6_	239.0557	3.38	−0.717	1, 2, 3	2
**8.**	caffeoyl-syringic acid	C_18_H_16_O_8_	359.0985	2.32	0.390	1, 2, 3	4
**Hydroxybenzoic and hydroxycinnamc acids glycosides**							
**9.**	4-hydroxyphenylacetic acid *O-*β-D-glucoside	C_14_H_18_O_8_	313.0933	2.18	1.467	2	2
**10.**	vanillic acid *O*-deoxyhexoside	C_14_H_18_O_8_	313.0934	3.25	1.467	2, 3	2
**11.**	gentisic acid *O*-hexoside	C_14_H_20_O_8_	315.1087	1.92	0.601	1, 2, 3	2
**12.**	*p*-hydroxybenzoic acid *O*-hexoside	C_14_H_20_O_8_	315.1086	2.10	0.029	1, 2, 3	2
**13.**	vanillic acid *O*-hexoside	C_14_H_18_O_9_	329.0885	1.71	2.020	1, 2, 3	2
**14.**	leonuriside A	C_14_H_20_O_9_	331.1037	1.44	0.739	1, 2, 3	2
**15.**	gallic acid *O*-hexoside	C_13_H_16_O_10_	331.0676	1.58	1.601	2	2
**Acylquinic acids**							
**16.**	1-*p*-coumaroylquinic acid	C_16_H_18_O_8_	337.0932	4.61	1.007	1, 2	2
**17.**	3-*p*-coumaroylquinic acid	C_16_H_18_O_8_	337.0935	3.01	1.748	2	2
**18.**	1-caffeoylquinic acid	C_16_H_18_O_9_	353.0880	2.27	0.410	1, 2	2
**19.**	neochlorogenic (3-caffeoylquinic) acid	C_16_H_18_O_9_	353.0878	3.21	−0.015	1, 2, 3	1
**20.**	chlorogenic (5-caffeoylquinic) acid ^a^	C_16_H_18_O_9_	353.0874	3.94	−1.233	1, 2, 3	1
**21.**	4-caffeoylquinic acid	C_16_H_18_O_9_	353.0879	6.27	0.155	1, 2, 3	2
**22.**	3,4-dicaffeoylquinic acid ^a^	C_25_H_24_O_12_	515.1199	5.73	0.836	1, 2, 3	1
**23.**	1,5-dicaffeoylquinic acid ^a^	C_25_H_24_O_12_	515.1191	5.91	−0.697	1, 2, 3	1
**24.**	3,5-dicaffeoylquinic acid	C_25_H_24_O_12_	515.1199	6.08	0.720	1, 2, 3	1
**25.**	4,5-dicaffeoylquinic acid	C_25_H_24_O_12_	515.1191	6.25	−0.697	1, 2, 3	1
**26.**	1,3,5-tricaffeoylquinic acid	C_34_H_30_O_15_	677.1512	5.15	-	1, 2, 3	1
**Flavonoids**							
**27.**	apigenin ^a^	C_15_H_9_O_5_	269.0459	8.58	1.313	1, 3	1
**28.**	genkwanin ^a^	C_16_H_12_O_5_	283.0608	11.41	−1.543	2	1
**29.**	acacetin	C_16_H_12_O_5_	283.0615	11.40	1.142	1, 3	2
**30.**	luteolin ^a^	C_15_H_10_O_6_	285.0404	7.55	−0.075	1, 3	1
**31.**	hispidulin (scutellarein-6-methyl ether) ^a^	C_16_H_12_O_6_	299.0561	8. 81	−0.172	1, 2, 3	1
**32.**	diosmetin	C_16_H_12_O_6_	299.0560	9.28	−0.272	1	1
**33.**	quercetin ^a^	C_15_H_9_O_6_	301.0354	7.61	1.11	1	1
**34.**	pectolinarigenin	C_17_H_14_O_6_	313.0722	12.26	1.305	1, 2, 3	2
**35.**	nepetin (6-methoxyluteolin)	C_16_H_11_O_7_	315.0514	8.09	1.251	1, 3	2
**36.**	cirsiliol	C_17_H_14_O_7_	329.0669	8.87	0.772	1	2
**37.**	apigenin 7-*O*-glucoside ^a^	C_21_H_20_O_10_	431.0988	6.06	0.835	1	1
**38.**	kaempferol 3-*O*-deoxyhexoside	C_21_H_20_O_10_	431.0983	6.60	−0.232	1, 2	2
**39.**	apigenin *O*-hexuronide	C_21_H_18_O_11_	445.0770	6.45	−0.347	1, 2, 3	2
**40.**	kaempferol 3-*O*-glucoside ^a^	C_21_H_20_O_11_	447.0935	5.63	0.571	1, 2	1
**41.**	luteolin 7-*O*-glucoside ^a^	C_21_H_19_O_11_	447.0934	6.04	0.281	1, 2, 3	1
**42.**	luteolin 7-*O*-hexuronide	C_21_H_18_O_12_	461.0734	5.37	1.911	1, 3	2
**43.**	diosmetin 7-*O*-hexoside	C_22_H_22_O_11_	461.1092	6.30	0.684	1, 2, 3	2
**44.**	hispidulin 7-*O*-hexoside	C_22_H_22_O_11_	461.1093	6.67	0.966	1, 2, 3	2
**45.**	hispidulin-*O*-hexuronide	C_22_H_20_O_12_	475.0882	6.33	0.002	1, 3	2
**46.**	pectolinarigenin-*O*-hexoside	C_23_H_24_O_11_	475.1247	8.11	0.159	1	2
**47.**	nepetin-*O*-hexoside	C_22_H_21_O_12_	477.1040	5.65	0.316	1, 3	2
**48.**	nepetin-*O*-hexuronide	C_22_H_20_O_13_	491.0835	6.32	0.725	1	2
**49.**	acaciin (acacetin 7-*O*-rutinoside) ^a^	C_28_H_32_O_14_	591.1730	7.59	3.622	1, 2, 3	1
**50.**	kaempferol 3-*O*-rutinoside ^a^	C_27_H_30_O_15_	593.1532	5.40	3.383	1, 3	1
**51.**	hispidulin 7-*O*-rutinoside	C_28_H_32_O_15_	607.1675	6.34	1.049	1, 2, 3	2
**52.**	pectolinarin (pectolinarigenin 7-*O*- rutinoside) ^a^	C_29_H_34_O_15_	621.1824	7.67	−0.199	1, 2, 3	1
**Free fatty acids**							
**53.**	nonanedioic acid (azelaic acid)	C_9_H_16_O_4_	187.0967	6.32	−4.502	1, 2, 3	2
**54.**	3-hydroxysuberic acid	C_8_H_14_O_5_	189.0758	4.64	−5.483	1, 2, 3	2
**55.**	3-hydroxyazelaic acid	C_9_H_16_O_5_	203.0918	6.25	−3.677	1, 2, 3	2
**56.**	2-dodecenoic acid	C_12_H_20_O_4_	227.1287	9.46	−0.715	2, 3	2
**57.**	9,13-dyhidroxyoctadeca-9,11,13-trienoic acid	C_18_H_30_O_4_	309.2074	12.76	0.768	2, 3	2
**58.**	11,12-dyhidroxyoctadeca-9,13,15-trienoic acid	C_18_H_30_O_4_	309.2075	12.91	−0.332	2	2
**59.**	9,10-dyhidroxyoctadeca-12,14,16-trienoic acid	C_18_H_30_O_4_	309.2074	10.81	1.835	2	2
**60.**	9,13-dyhidroxyoctadeca-11,13-dienoic acid	C_18_H_32_O_4_	311.2231	13.67	0.859	1, 2, 3	2
**61.**	9,10-dyhidroxyoctadeca-9-enoic acid	C_18_H_34_O_4_	313.2388	13.79	0.885	3	2

^a^ Compare to reference standards. 1–flower heads; 2–aerial parts; 3–roots.

**Table 2:** Total bioactive compounds and antioxidant properties of *Cirsium appendiculatum* extracts *.

Parts	Total Phenolic Content (mgGAE/g)	Total Flavonoid Content (mgRE/g)	DPPH^•^ (mgTE/g)	ABTS^•+^ (mgTE/g)	CUPRAC (mgTE/g)	FRAP (mg TE/g)	PHMD (mmolTE/g)	Metal Chelating (mgEDTAE/g)
Flower heads	71.75 ± 1.47 ^b^	46.59 ± 0.89 ^a^	101.79 ± 0.15 ^a^	224.57 ± 0.57 ^a^	356.97 ± 11.52 ^b^	169.60 ± 0.84 ^b^	1.71 ± 0.07 ^b^	32.53 ± 3.51 ^a^
Aerial parts	26.02 ± 1.49 ^c^	2.64 ± 0.08 ^c^	70.25 ± 1.91 ^c^	124.16 ± 4.73 ^b^	103.77 ± 5.89 ^c^	69.98 ± 2.01 ^c^	0.74 ± 0.01 ^c^	9.42 ± 0.54 ^b^
Roots	143.62 ± 2.99 ^a^	3.99 ± 0.06 ^b^	97.95 ± 0.60 ^b^	224.59 ± 0.33 ^a^	618.36 ± 5.17 ^a^	269.89 ± 8.50 ^a^	3.36 ± 0.15 ^a^	na

* Values are expressed as mean ± S.D (*n*: 3). GAE: Gallic acid equivalent; RE: Rutin equivalent; TE: Trolox equivalent; EDTAE: EDTA equivalent; na: not active. Different letters (a, b and c) indicate significant differences in the extracts (*p* < 0.05)

**Table 3:** Enzyme inhibitory properties of *Cirsium appendiculatum* extracts *.

Parts	AChE Inhibition (mgGALAE/g)	BChE Inhibition (mgGALAE/g)	Tyrosinase (mgKAE/g)	Amylase (mmolACAE/g)	Glucosidase (mmolACAE/g)
Flower heads	4.40 ± 0.40 ^a^	1.54 ± 0.07 ^c^	97.78 ± 0.76 ^c^	0.60 ± 0.01 ^a^	0.41 ± 0.10 ^b^
Aerial parts	3.52 ± 0.31 ^b^	2.67 ± 0.34 ^b^	110.61 ± 0.79 ^b^	0.61 ± 0.06 ^a^	na
Roots	4.93 ± 0.25 ^a^	3.80 ± 0.26 ^a^	127.99 ± 0.68 ^a^	0.62 ± 0.04 ^a^	0.72 ± 0.07 ^a^

* Values are expressed as mean ± S.D (*n*: 3). GALAE: Galatamine equivalent; KAE: Kojic acid equivalent; ACAE: Acarbose equivalent; na: not active. Different letters (a, b and c) indicate significant differences in the extracts (*p* < 0.05)

## Data Availability

Not applicable.

## Supplementary Materials

The following are available online at www.mdpi.com/article/10.3390/plants10102046/s1, Table S1: Specialized natural products in Cirsium appendiculatum extracts; Figure S1: Variation of identified natural products between the Cirsium appendiculatum organs (flower heads, aerial parts and roots).
