# Supplementary material for: Innovative Biochemometric Approach to the Metabolite and Biological Profiling of the Balkan Thistle (Cirsium appendiculatum Griseb.), Asteraceae
Source: Plants (Basel). 2021 Sep 28;10(10):2046. doi: 10.3390/plants10102046 (PMC8539897; doi:10.3390/plants10102046)
Supplement: Supplementary file 1 [file plants-10-02046-s001.zip › plants-1372179-supplementary.pdf]

Article

Innovative biochemometric approach to the metabolite and biological profiling of the Balkan thistle (*Cirsium appendiculatum* Griseb.), Asteraceae

Dimitrina Zheleva-Dimitrova[1*], Gokhan Zengin[2], Gunes Ak[2], Kouadio Ibrahime Sinan[2], Mohamad Fawzi Mahomoodally[3], Reneta Gevrenova[1], Vessela Balabanova[1], Alexandra Stefanova[1], Paraskev Nedialkov[1], Yulian Voynikov[4]

[1] Department of Pharmacognosy, Faculty of Pharmacy, Medical University-Sofia, 2 Dunav str., Sofia 1000, Bulgaria dzheleva@pharmfac.mu-sofia.bg (D.Z-D.); rgevrenova@pharmfac.mu-sofia.bg (R.G.); vbalabanova@pharmfac.mu-sofia.bg (V.B.); al_stefanova@abv.bg (A.S.); pnedialkov@pharmfac.mu-sofia.bg (P.N.)
[2] Biochemistry and Physiology Research Laboratory, Department of Biology, Science Faculty, Selcuk University, Campus, Konya, Turkey gokhanzengin@selcuk.edu.tr (G.Z.); akguneselcuk@gmail.com (G.A.); sinankouadio@gmail.com (K.I.S.);
[3] Department of Health Sciences, Faculty of Medicine and Health Sciences, University of Mauritius, Réduit, Mauritius f.mahomoodally@uom.ac.mu (M.F.M.)
[4] Department of Chemistry, Faculty of Pharmacy, Medical University-Sofia, Bulgaria 2 Dunav str., Sofia 1000, Bulgaria voynikov_y@pharmfac.mu-sofia.bg (Y.V.)
* Correspondence: dzheleva@pharmfac.mu-sofia.bg; Tel.: +359898254383

Table S1. Specialized natural products in *Cirsium appendiculatum* extracts

№	Identified/tentatively annotated compound	Molecular formula	Exact mass [M-H]$^-$	Fragmentation pattern in (-) ESI-MS/MS	t_R (min)	Δ ppm	Distribution	Level of identification (CAWG)
				**Carboxylic, hydroxybenzoic and hydroxycinnamic acids**				
**1.**	protocatechuic acid[a]	$C_7H_6O_4$	153.0179	153.0184 (12), 109.0281 (100)	2.15	-7.986	1,2	1
**2.**	dihydroxybenzoic acid	$C_7H_6O_4$	153.0181	153.0181 (54.79), 123.0074 (100), 109.0283 (20.48)	3.44	-8.182	2,3	2
**3.**	gentisic acid[a]	$C_7H_6O_4$	153.0179	153.0183 (54), 135.0075 (32), 109.0281 (100), 91.0175 (8)	3.86	-9.685	2	1
**4.**	vanillic acid[a]	$C_8H_8O_4$	167.0338	167.0341 (100), 152.0105 (28), 124.0154 (24), 111.0075 (12), 95.0123 (9)	4.77	-6.837	1,2,3	1
**5.**	caffeic acid[a]	$C_9H_8O_4$	179.0340	179.0342 (12), 135.0439 (100), 107.0489 (1)	3.51	-5.317	1,2,3	1
**6.**	quinic acid	$C_7H_{12}O_6$	191.0551	191.0554 (100), 173.0445 (2), 127.0388 (5), 111.0437 (3), 93.0331 (11), 85.0280 (31)	3.18	-5.032	1,2,3	2
**7.**	eucomic acid	$C_{11}H_{12}O_6$	239.0557	239.0559 (18.35), 221.0459 (0.59), 195.0658 (7.18), 179.0341 (100), 177.0549 (61.93), 149.0596 (46.80), 133.0645 (16.95), 107.0488 (34.43)	3.38	-0.717	1,2,3	2
**8.**	caffeoyl-syringic acid	$C_{18}H_{16}O_8$	359.0985	359.0971 (7.95), 197.0448 (100), 182.0213 (20.40), 166.9974 (6.15), 153.0546 (13.36), 123.0073 (32.61)	2.32	0.390	1,2,3	4
				**Hydroxybenzoic and hydroxycinnamc acids glycosides**				
**9.**	4- hydroxyphenylacetic acid *O*-*β-D*-glucoside	$C_{14}H_{18}O_8$	313.0933	313.0914 (5.86),151.0388 (100), 123.0434 (15.72), 107.0485 (24.86), 89.0228 (3.17)	2.18	1.467	2	2
**10.**	vanillic acid *O*-deoxyhexoside	$C_{14}H_{18}O_8$	313.0934	313.0924 (17.72), 167.0341 (63.33), 152.0102 (18.47), 151.0388 (17.57), 123.0435 (27.04), 107.0487 (100), 97.4587 (11.11)	3.25	1.467	2,3	2
**11.**	gentisic acid *O*-hexoside	$C_{14}H_{20}O_8$	315.1087	315.1087 (4.79), 153.0544 (100), 123.0437 (50.06)	1.92	0.601	1,2,3	2
**12.**	*p*-hydroxybenzoic acid *O*-hexoside	$C_{14}H_{20}O_8$	315.1086	315.1089 (100), 153.0544 (38.59), 135.0438 (74.52)	2.10	0.029	1,2,3	2
**13.**	vanillic acid *O*-hexoside	$C_{14}H_{18}O_9$	329.0885	329.0877 (1.89), 167.0337 (100), 152.0102 (21.76), 123.0436 (15.76), 108.0200 (38.79)	1.71	2.020	1,2,3	2

**14.**	leonuriside A	$C_{14}H_{20}O_9$	331.1037	331.1034 (17.78), 300.8454 (0.97), 169.0493 (16.52), 168.0417 (80.32), 167.0339 (13.12), 153.0181 (100), 137.9945 (12.07), 109.9994 (9.92)	1.44	0.739	1,2,3	2
**15.**	gallic acid *O*-glucoside	$C_{13}H_{16}O_{10}$	331.0676	331.0669 (8.48), 169.0132 (100), 153.0180 (2.18), 125.0231 (42.85)	1.58	1.601	2	2
				**Acylquinic acids**				
**16.**	1-*p*-coumaroylquinic acid	$C_{16}H_{18}O_8$	337.0932	337.0912 (7.55), 191.0552 (100), 173.0440 (6.75), 93.0330 (17.57)	4.61	1.007	1,2	2
**17.**	3-*p*-coumaroylquinic acid	$C_{16}H_{18}O_8$	337.0935	337.0925 (7.69), 191.0555 (8.10), 173.0443 (3.38), 163.0388 (100), 135.0435 (0.68)	3.01	1.748	2	2
**18.**	1-caffeoylquinic acid	$C_{16}H_{18}O_9$	353.0880	353.0887 (32.77), 191.0551 (100), 179.0340 (54.69), 161.0224 (3.41), 135.0438 (41.62), 93.0331 (4.71), 85.0280 (9.10)	2.27	0.410	1,2	2
**19.**	neochlorogenic (3-caffeoylquinic) acid	$C_{16}H_{18}O_9$	353.0878	353.0882 (41.32), 191.0552 (100), 179.0340 (63.89), 173.0445 (3.07),161.0235 (3.39), 135.0438 (48.77), 93.0331 (4.38), 85.0280 (8.05)	3.21	-0.015	1,2,3	1
**20.**	chlorogenic (5-caffeoylquinic) acid[a]	$C_{16}H_{18}O_9$	353.0874	353.0874 (3.83), 191.0551 (100), 179.0338 (1.27), 161.0229 (1.31), 93.0330 (2.76), 85.0279 (7.63)	3.94	-1.233	1,2,3	1
**21.**	4-caffeoylquinic acid	$C_{16}H_{18}O_9$	353.0879	353.0882 (32.08), 191.0552 (42.60), 179.0340 (68.86), 173.0444 (100), 135.0438 (50.21), 93.0330 (20.65), 85.0280 (8.08)	6.27	0.155	1,2,3	2
**22.**	3,4-dicaffeoylquinic acid[a]	$C_{25}H_{24}O_{12}$	515.1199	515.1190 (90.07), 353.0876 (64.15), 335.0786 (7.05), 203.0340 (44.63), 191.0555 (37.66), 179.0337 (70.78), 173.0446 (100), 161.0234 (8.34), 135.0437 (83.40), 93.0329 (23.86), 85.0278 (6.31)	5.73	0.836	1,2,3	1
**23.**	1,5-dicaffeoylquinic acid[a]	$C_{25}H_{24}O_{12}$	515.1191	515.1212 (17.11), 353.0883 (88.58), 191.0551 (100), 179.0340 (33.86), 161.0231 (5.38), 135.0438 (32.71), 93.0330 (3.44), 85.0278 (7.21)	5.91	-0.697	1,2,3	1
**24.**	3,5-dicaffeoylquinic acid	$C_{25}H_{24}O_{12}$	515.1199	515.1216 (20.12), 353.0883 (100), 191.0553 (98.44), 179.0340 (52.77), 173.0442 (5.24),	6.08	0.720	1,2,3	1

				135.0438 (52.22), 93.0329 (2.62), 85.0278 (7.98)				
**25.**	4,5-dicaffeoylquinic acid	$C_{25}H_{24}O_{12}$	515.1191	515.1206 (93.50), 353.0886 (57.84), 335.0781 (1.46), 191.0551 (40.57), 179.0339 (68.33), 173.0444 (100), 161.0234 (3.07), 135.0437 (66.12), 93.0329 (24.19), 85.0279 (3.33)	6.25	-0.697	1,2,3	1
**26.**	1,3,5-tricaffeoylquinic acid	$C_{34}H_{30}O_{15}$	677.1512	515.1404 (23.3), 353.0889 (21.2), 341.0884 (21.1), 323.0765 (42.2), 191.0555 (100), 179.0339 (47.7), 173.0447 (3.4), 161.0235 (43.0), 135.0440 (62.3), 93.0330 (7.8), 85.0279 (18.7)	5.15	-	1,2,3	1
				**Flavonoids**				
**27.**	apigenin[a]	$C_{15}H_{9}O_{5}$	269.0459	269.0455 (100), 225.0674 (3.15), 151.0021 (5.17), 117.0332 (15.77), 107.0121 (2.98)	8.58	1.313	1,3	1
**28.**	genkwanin[a]	$C_{16}H_{12}O_{5}$	283.0608	283.0610 (100), 268.0376 (0.41), 240.0419 (4.90), 239.0348 (3.73), 211.0399 (1.09), 171.0434 (0.92), 165.3494 (0.55), 151.0020 (2.06), 117.0333 (0.97), 107.0126 (0.92)	11.41	-1.543	2	1
**29.**	acacetin	$C_{16}H_{12}O_{5}$	283.0615	283.0610 (100), 268.0376 (62.88), 240.0423 (4.43), 239.0344 (4.15), 171.0452 (2.35), 151.0025 (3.07), 107.0123 (2.82)	11.40	1.142	1,3	2
**30.**	luteolin[a]	$C_{15}H_{10}O_{6}$	285.0404	285.0404 (100), 175.0396 (2.60), 151.0026 (2.93), 133.0279 (20.14), 107.0115 (1.59)	7.55	-0.075	1,3	1
**31.**	hispidulin (scutellarein-6-methyl ether)[a]	$C_{16}H_{12}O_{6}$	299.0561	299.0564 (69.86), 285.0349 (10.96), 284.0327 (100), 228.0403 (2.41), 212.0464 (2.70), 136.9863 (14.52), 227.0329 (2.24), 117.0326 (1.99)	8. 81	-0.172	1,2,3	1
**32.**	diosmetin	$C_{16}H_{12}O_{6}$	299.0560	299.0563 (61.78), 285.0362 (8.05), 284.0327 (100), 256.0359 (4.76), 255. 0298 (45.54), 227.0345 (51.09), 211.0379 (1.86), 183.0465 (1.50), 151.0026 (0.45), 107.0124 (0.39)	9.28	-0.272	1	1
**33.**	quercetin[a]	$C_{15}H_{9}O_{6}$	301.0354	301.0355 (100), 273.0398 (1.71), 178.9979 (20.42), 151.0024 (40.30), 121.0283 (11.37), 107.0125 (11.01)	7.61	1.11	1	1
**34.**	pectolinarigenin	$C_{17}H_{14}O_{6}$	313.0722	313.0719 (92.49), 298.0484 (100), 283.0250 (53.19), 255.0299 (21.20), 227.0343 (7.14),	12.26	1.305	1,2,3	2

				211.0391 (2.18), 183.0442 (4.31), 163.0024 (17.83), 135.0010 (96.43), 117.0332 (15.12)				
**35.**	nepetin (6-methoxyluteolin)	$C_{16}H_{11}O_7$	315.0514	315.0510 (96.43), 300.0274 (100), 136.9866 (13.77), 271.0255 (8.97), 255.0309 (4.29), 227.0348 (4.39), 133.0284 (2.59)	8.09	1.251	1,3	2
**36.**	cirsiliol	$C_{17}H_{14}O_7$	329.0669	329.0667 (100), 314.0435 (66.77), 299.0199 (41.90), 271.0251 (28.65), 227.0350 (16.01), 203.0341 (8.50), 161.0234 (18.12), 151.0029 (1.69), 116.8058 (1.55)	8.87	0.772	1	2
**37.**	apigenin 7-*O*-glucoside[a]	$C_{21}H_{20}O_{10}$	431.0988	431.0984 (100), 269.0447 (25.89), 268.0375 (61.00), 211.0394 (1.80), 151.0023 (3.98), 117.0328 (1.88), 107.0122 (2.25)	6.06	0.835	1	1
**38.**	kaempferol 3-*O*-deoxyhexoside	$C_{21}H_{20}O_{10}$	431.0983	431.0994 (100), 285.0402 (65.76), 284.0327 (43.66), 255.0298 (35.47), 227.0349 (31.45), 171.3882 (5.68), 116. 1093 (5.53)	6.60	-0.232	1,2	2
**39.**	apigenin *O*-hexuronide	$C_{21}H_{18}O_{11}$	445.0770	445.0776 (29.58), 269.0455 (100), 175.0230 (13.50), 117.0331 (6.66), 113.0230 (0.92), 151.0026 (1.30), 107.0125 (3.18)	6.45	-0.347	1,2,3	2
**40.**	kaempferol 3-*O*-glucoside[a]	$C_{21}H_{20}O_{11}$	447.0935	447.0941 (100), 403.7003 (2.72), 285.0461 (5.58), 284.0324 (58.94), 255.0297 (36.40), 227.0344 (44.91), 145.9196 (2.95)	5.63	0.571	1,2	1
**41.**	luteolin 7-*O*-glucoside[a]	$C_{21}H_{19}O_{11}$	447.0934	447.0945 (100), 285.0406 (48.48), 284.0326 (28.16), 257.0456 (8.51), 229.1260 (1.88)151.0023 (21.51), 107. 0123 (15.12)	6.04	0.281	1,2,3	1
**42.**	luteolin 7-*O*-hexuronide	$C_{21}H_{18}O_{12}$	461.0734	461.0726 (55.43), 285.0403 (100), 211.0396 (0.82), 243.0296 (0.60), 151.0020 (4.58), 133.0278 (8.59), 107.0122 (2.83)	5.37	1.911	1,3	2
**43.**	diosmetin 7-*O*-hexoside	$C_{22}H_{22}O_{11}$	461.1092	461.1089 (100), 446.0850 (35.75), 299.0559 (24.09), 283.0247 (98.56), 255.0296 (65.21), 227.0348 (4.39), 183.0442 (5.70), 151.0024 (1.78)	6.30	0.684	1,2,3	2
**44.**	hispidulin 7-*O*-hexoside	$C_{22}H_{22}O_{11}$	461.1093	461.1089 (100), 299.0553 (9.22), 298.0486 (10.58), 283.0247 (34.29), 255.0297 (39.69), 227.0348 (3.47), 183.0443 (1.46), 163.0030 (6.59), 117.0331 (6.54)	6.67	0.966	1,2,3	2
**45.**	hispidulin-*O*-hexuronide	$C_{22}H_{20}O_{12}$	475.0882	475.0874 (60.01), 355.0584 (2.41), 300.0589 (5.61), 299.0558 (100), 284.0322 (65.59),	6.33	0.002	1,3	2

				251.2390 (2.41), 175.0239 (14.39), 113.0230 (29.07)				
**46.**	pectolinarigenin-*O*-hexoside	$C_{23}H_{24}O_{11}$	475.1247	475.1248 (96.00), 313.0720 (100), 298.0480 (46.59), 297.0406 (80.31), 283.0252 (26.27), 269.0460 (28.90), 254.0217 (17.13), 183.9528 (3.13), 163.0028 (4.03)	8.11	0.159	1	2
**47.**	nepetin-*O*-hexoside	$C_{22}H_{21}O_{12}$	477.1040	477.1034 (100), 315.0509 (36.29), 314.0435 (7.71), 313.0352 (10.25), 301.0337 (3.01), 299.0201 (26.36), 271.0253 (3.11), 227.0351 (4.92), 199.0394 (9.36), 163.0027 (1.61), 164.9815 (1.22), 151.1239 (1.18), 133.0279 (13.36)	5.65	0.316	1,3	2
**48.**	nepetin-*O*-hexuronide	$C_{22}H_{20}O_{13}$	491.0835	491.0832 (76.47), 315.0512 (100), 300.0277 (78.63), 285.2417 (3.66), 165.9894 (5.71), 133.0239 (3.67)	6.32	0.725	1	2
**49.**	acaciin (acacetin 7-*O*-rutinoside)[a]	$C_{28}H_{32}O_{14}$	591.1730	591.1730 (14.41), 285.3700 (0.45), 284.0645 (10.40), 283.0609 (100), 269.0405 (5.36), 269.0375 (41.91), 240.0408 (0.43), 151.0027 (0.43), 163.7786 (0.19)	7.59	3.622	1,2,3	1
**50.**	kaempferol 3-*O*-rutinoside[a]	$C_{27}H_{30}O_{15}$	593.1532	593.1527 (100), 285.0401 (30.14), 284.0328 (62.52), 255.0299 (37.88), 227.0346 (23.78), 211.0395 (1.01), 183.0447 (0.42), 151.0022 (2.09), 163.0031 (0.74), 135.0078 (0.90), 117.0332 (0.32), 107.0124 (1.10)	5.40	3.383	1,3	1
**51.**	hispidulin 7-*O*-rutinoside	$C_{28}H_{32}O_{15}$	607.1675	607.1670 (27.44), 299.0560 (100), 284.0325 (19.84), 255.0297 (32.62), 227.0344 (25.33), 211.0393 (0.70), 183.0436 (0.21)	6.34	1.049	1,2,3	2
**52.**	pectolinarin (pectolinarigenin 7-*O*- rutinoside)[a]	$C_{29}H_{34}O_{15}$	621.1824	621.1873 (0.86), 313.0716 (100), 298.0482 (27.54), 283.0247 (27.97), 285.3503 (0.33), 283.0247 (27.97), 269.0455 (0.49), 255.0296 (7.06), 227.0346 (1.83), 163.0024 (3.88), 117.033 (2.09)	7.67	-0.199	1,2,3	1
				**Free fatty acids**				
**53.**	nonanedioic acid (azelaic acid)	$C_9H_{16}O_4$	187.0967	187.0968 (46.19), 169.0862 (4.11), 143.1065 (13.05), 125.0958 (100), 97.0643 (6.97)	6.32	-4.502	1,2,3	2

**54.**	3-hydroxysuberic acid	$C_8H_{14}O_5$	189.0758	189.60 (18.68), 171.0652 (4.32), 145.0858 (4.17), 129.0544 (100), 127.0751 (27.17), 101.0591 (0.78), 99.0800 (20.31), 85.0280 (0.78), 59.0123 (1.89)	4.64	-5.483	1,2,3	2
**55.**	3-hydroxyazelaic acid	$C_9H_{16}O_5$	203.0918	203.0917 (20.10), 185.0810 (5.59), 159.1014 (6.23), 143.0700 (100), 141.0907 (36.68), 115.0753 (0.18), 113.0957 (29.80), 71.0534 (0.17), 59.0122 (2.13)	6.25	-3.677	1,2,3	2
**56.**	2-dodecenoic acid	$C_{12}H_{20}O_4$	227.1287	227.1286 (17.41), 183.1382 (100), 165.1273 (15.25)	9.46	-0.715	2,3	2
**57.**	9,13-dyhidroxyoctadeca-9,11,13-trienoic acid	$C_{18}H_{30}O_4$	309.2074	309.2074 (56.01), 291.1972 (27.01), 268.9843 (10.38), 225.1493 (9.30), 209.1540 (7528), 197.1175 (17.00), 185.1173 (2.98), 171.1019 (20.40), 151.0754 (11.64), 99.0071 (100), 701617 (36.14)	12.76	0.768	2,3	2
**58.**	11,12-dyhidroxyoctadeca-9,13,15-trienoic acid	$C_{18}H_{30}O_4$	309.2075	309.2086 (3.81), 291.1966 (15.71), 273.1854 (2.31), 263.2018 (4.55), 247.2071 (1.58), 230.9864 (1.28), 211.1333 (14.32), 197.1176 (100), 169.1223 (14.79), 153.1273 (2.43), 127.0756 (2.30), 111.0800 (44.34), 97.0643 (1.28)	12.91	-0.332	2	2
**59.**	9,10-dyhidroxyoctadeca-12,14,16-trienoic acid	$C_{18}H_{30}O_4$	309.2074	309.2071 (100), 291.1966 (59.98), 247.2070 (1.89), 209.1175 (0.39), 193.1218 (0.41), 171.1015 (52.29), 137.0958 (19.12)	10.81	1.835	2	2
**60.**	9,13-dyhidroxyoctadeca-11,13-dienoic acid	$C_{18}H_{32}O_4$	311.2231	311.2231 (100), 293.2123 (44.45), 211.1334 (43.08), 185.1174 (19.67), 171.1016 (65.58), 155.1062 (6.88), 139.1115 (22.16), 129.0907 (12.59), 99.0801 (5.90)	13.67	0.859	1,2,3	2
**61.**	9,10-dyhidroxyoctadeca-9-enoic acid	$C_{18}H_{34}O_4$	313.2388	313.2387 (100), 295.2278 (13.55), 277.2173 (11.92), 201.1125 (11.52), 183.1022 (1.84), 171.1017 (10.37)	13.79	0.885	3	2

[a]-Compare to reference standards
1-flower heads; 2-aerial parts; 3-roots

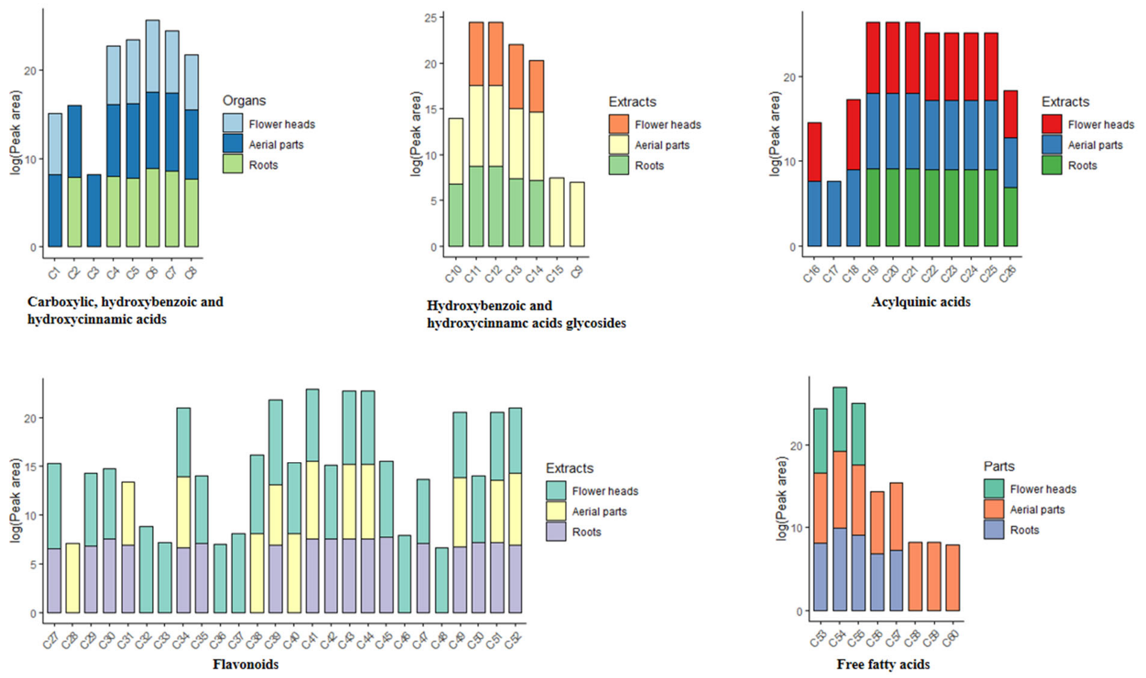

Figure S1. Variation of identified natural products between the *Cirsium appendiculatum* organs (flower heads, aerial parts and roots)
